# Comparable Outcomes and Health-Related Quality of Life for Severe Aplastic Anemia: Haploidentical Combined With a Single Cord Blood Unit *vs* Matched Related Transplants

**DOI:** 10.3389/fonc.2021.714033

**Published:** 2022-01-18

**Authors:** Meiqing Lei, Xiaoli Li, Yanming Zhang, Qi Qu, Wenjing Jiao, Huifen Zhou, Qingyuan Wang, Huiying Qiu, Xiaowen Tang, Yue Han, Chengcheng Fu, Zhengming Jin, Suning Chen, Aining Sun, Miao Miao, Limin Liu, Depei Wu

**Affiliations:** ^1^ National Clinical Research Center for Hematologic Diseases, Jiangsu Institute of Hematology, The First Affiliated Hospital of Soochow University, Institute of Blood and Marrow Transplantation of Soochow University, Suzhou, China; ^2^ Department of Hematology, Haikou Municipal People’s Hospital, Affiliated Haikou Hospital of Xiangya Medical College, Central South University, Haikou, China; ^3^ Soochow Hopes Hematonosis Hospital, Suzhou, China; ^4^ Department of Hematology, The Affiliated Huai’an Hospital of Xuzhou Medical University and the Second People’s Hospital of Huai’an, Huai’an, China; ^5^ Department of Hematology, Xian Yang Central Hospital, Xianyang, China

**Keywords:** severe aplastic anemia, transplantation, haploidentical donor, matched related donor, unrelated cord blood, health-related quality of life

## Abstract

We retrospectively compared the outcomes and health-related quality of life (HRQoL) of severe aplastic anemia (SAA) patients who received haploidentical hematopoietic stem cell transplantation with a single unrelated cord blood unit (Haplo-cord HSCT) (n = 180) or matched related donor (MRD)-HSCT (n = 128). After propensity score matching, we were able to match 88 patients in each group and to compare the outcomes between the two matched-pair groups. Haplo-cord recipients exhibited a longer median days for neutrophil engraftment (12 *vs* 11, *P* = 0.001) and for platelet engraftment (15 *vs* 13, *P* = 0.003). Haplo-cord recipients a high cumulative incidence of grades II–IV acute graft-versus-host disease (GVHD) (29.8 *vs* 14.0%, *P* = 0.006), while similar III–IV acute GVHD, total chronic GVHD, and moderate to severe chronic GVHD at four-year (all *P* < 0.05). Among the Haplo-cord HSCT and MRD-HSCT groups, the four-year GVHD-free/failure-free survival rates were 73.5% and 66.9% (*P* = 0.388) respectively, and the overall survival rates were 81.5% and 77.2% (*P* = 0.484), respectively. Similar comparable results also were observed between the corresponding first-line, older or younger than 40 years old subgroups. The Haplo-cord HSCT group exhibited higher scores in the physical component summary, physical functioning, general health and social functioning than the MRD-HSCT group (all *P* < 0.05). In the multivariate analysis, young age and Haplo-cord HSCT were favorable factors for HRQoL, while moderate to severe cGVHD was associated with lower HRQoL. These results suggest that for SAA patients, Haplo-cord HSCT could achieve at least comparable efficacy and HRQoL to MRD-HSCT.

## Introduction

Acquired severe aplastic anemia (SAA) is a kind of bone marrow (BM) failure syndromes mainly caused by immune destruction of hemopoietic stem and progenitor cells (1, 2). For SAA, including very SAA (vSAA), once diagnosed, individuals require effective treatment as soon as possible. Otherwise, it may be life-threatening due to severe bleeding and infection. The choice of treatment for SAA is determined based on patient age, donor availability, and access to therapeutic resources. According to published guidelines, human leukocyte antigen (HLA)-matched related donor (MRD) transplantation is the preferred option for young SAA patients. However, immunosuppressive therapy (IST) using antithymocyte globulin (ATG) and cyclosporine A (CsA) is indicated for young patients who do not have a MRD and patients older than 40 years. Haploidentical-HSCT (Haplo-HSCT) has been regarded as a salvage therapy when patients fail to respond to IST (3, 4). With continued progress in transplantation techniques, the age limit of allo-HSCT in SAA patients has been cautiously expanded to 50 years of age or older (5, 6). More importantly, Haplo-HSCT has achieved survival rates comparable to MRD-HSCT for SAA patients (7). Based on these observations, the latest guideline from the Chinese Society of Hematology recommends Haplo-HSCT as the front-line treatment for SAA patients without a HLA-matched donor (8).

To further improve the efficacy of Haplo-HSCT, some experienced transplant centers have been exploring some strategies to optimize this transplant strategy, including Haplo-HSCT combined with mesenchymal stem cells (MSCs) or unrelated cord blood (CB) (9–12). Until recently, another study reported encouraging results of sequential transplantation of haploidentical stem cell and unrelated CB on ATG/post-transplantation cyclophosphamide (PTCY) basis in relapsed/refractory hematologic malignancies, possibly by preventing graft-versus-host-disease (GVHD) and anti-leukemia effect (13). Notably, Haplo-HSCT with a single unrelated CB infusion (Haplo-cord HSCT) has exhibited encouraging survival outcomes for SAA patients in our clinical center (12). Meanwhile, health-related quality of life (HRQoL) assessment could help understand the burden of disease, provide direction for future therapy, and evaluate the efficacy of treatment interventions (14, 15). Therefore, HRQoL should be considered an integral component in evaluating the medical outcome of any treatments for SAA patients (16). As we reported before, the first-line Heplo-cord HSCT achieved similar overall survival (OS) and better failure-free survival (FFS) and HRQoL with the first-line IST for SAA patients (17). However, no direct comparison was performed including the HRQoL in SAA patients treated with Haplo-cord HSCT and MRD-HSCT. Thus, we performed this retrospective multicenter study to comprehensively compare the efficacy and the HRQoL between Haplo-cord HSCT and MRD-HSCT for SAA patients.

## Patients and Methods

### Patients

Between August 2003 and November 2019, 308 consecutive acquired SAA patients who underwent Haplo-cord HSCT (n = 180), or MRD-HSCT (n = 128) were enrolled in this study. Among the Haplo-cord HSCT group, previously reported 146 patients were also included (12). Inclusion criteria were as follows, (1) diagnosis of SAA (including vSAA) as defined by Camitta’s criteria (18), (2) transfusion was required, and (3) the presence of a relatively intact performance status and no apparent functional damage of internal organs (heart, liver, lung, and kidney) before transplantation. MRD-HSCT was the preferred choice for SAA patients, particularly those younger ones. Patients voluntarily participated in Haplo-HSCT in combination with unrelated UB infusion with the following circumstances, (a) without a matched related or unrelated donor, (b) refused to accept the first or second IST, (c) at least with one suitable haplo-identical donor (HID). The other exclusion criteria were as follows, patients with congenital bone marrow failure syndromes (Fanconi anemia, Diamond-Blackfan anemia, congenital dyskeratosis, and so on), patients who tested positive for myelodysplastic syndrome based on BM analyses, or diagnosed with other immunological diseases. Cytogenetic analyses of the BM and flow cytometry test of paroxysmal nocturnal hemoglobinuria (PNH) clone were routinely performed for all patients.

This study was carried out in accordance with the Declaration of Helsinki and approved by the participating hospitals’ Ethics Committees. All enrolled patients signed a written informed consent form prior to participation.

### Donor Selection

The HLA-A, -B, -C, DRB1, and -DQB1 typing of the recipients and donors, and the HLA-A, -B, and DRB1 typing of the unrelated CB units were performed. Donors were selected based on the HLA match, age, gender, health condition, and willingness to donate stem cells. Additional aspects concerning donor selection and the unrelated CB units were consistent with our previous report (12).

### Conditioning Regimen

The transplant days were numbered sequentially. The specific days preceding the transplant were indicated by a minus sign (−), such that the first day of the stem cell infusion was numbered “day 01,” the second day of infusion was “day 02”. The specific days after the last stem cell infusion were indicated by a plus sign (+). Patients in the Haplo-cord HSCT group were treated with a busulfan (BU)/cyclophosphamide (CY)-based regimen that included the following drugs. BU, 0.8 mg/kg intravenous (i.v.) was given four times daily on days −7 and −6. Cy, 50 mg/kg i.v., was given once daily from days −5 to −2, and ATG (rabbit, Thymoglobuline^®^, Genzyme, Cambridge, MA, USA), 2.5 mg/kg i.v., was given once daily from days −5 to −2. In the MRD-HSCT group, patients were given fludarabine (Flu) + CY + ATG regimen, which included Flu 30 mg/m^2^/day i.v. given for six days (days −7 to −2), Cy 50 mg/kg/day i.v. for two days (days −4 and −3), and ATG 2.5 mg/kg/day i.v., given for five days (days −8 to −4).

### Graft Collection and Infusion

From day −4 to the last day of stem cell collection, the hematopoietic stem cells from HIDs and MRDs were mobilized by subcutaneous injection of recombinant human granulocyte colony-stimulating factor (rhG-CSF) at a dose of 10 μg/kg/day. BM grafts from the MRDs were collected on day 01 *via* BM aspiration in the surgery room. The target mononuclear cells (MNCs) count from the BM was 6−8 × 10^8^/kg of recipient weight. If the target MNCs count was not achieved, peripheral blood stem cells (PBSCs) were collected the following day by apheresis using a COBE Spectra device (Gambro BCT, Lakewood, CO, USA). BM grafts from the HIDs were harvested on day 01 to attain a target MNCs count of 2–4 × 10^8^/kg of recipient weight, and PBSCs were collected the following day. The grafts from BM and peripheral blood (PB) were expected to provide the target MNCs count of 6–8 × 10^8^/kg of recipient weight. If the target count of cells was insufficient, additional PBSCs were collected on the following 1 to 2 days. Fresh unmanipulated BM and PBSCs were infused into the recipient on the day of collection. A single unrelated CB unit was infused 8 hour before the infusion of the Haploidentical grafts on day 01.

### GVHD Prophylaxis and Treatment

In the Haplo-cord HSCT group, CsA, mycophenolate mofetil (MMF), and short-term methotrexate (MTX) were administered for acute graft-versus-host-disease (aGVHD) prophylaxis (19). In the MRD-HSCT group, only CsA was used to prevent GVHD (beginning on day –4). Once GVHD occurred, the procedure of treatment was as described previously (12).

### Supportive Care and Post-Transplantation Surveillance

The details concerning supportive care and post-transplantation surveillance were consistent with previous experience (12).

### Definitions and Post-Transplantation Evaluations

Neutrophil engraftment was defined as the first day of an absolute neutrophil count (ANC) greater than 0.5 × 10^9^/L for three consecutive days. Platelet engraftment was defined as the first day of a platelet count greater than 20 × 10^9^/L for seven consecutive days without transfusion support. Primary graft failure (GF) was defined as failure to achieve neutrophil engraftment after HSCT up to + 28 days. Secondary GF was defined as recurrent pancytopenia with an ANC below 0.5 × 10^9^/L after a prior history of engraftment (20). Early mortality was defined as death within 60 days after HSCT. Transplantation-related mortality (TRM) was defined as death related to the transplantation and not the relapse of SAA. GVHD-free or failure-free survival (GFFS) was defined as survival without grade III–IV aGVHD, moderate to severe cGVHD, and treatment failures (including death, primary or secondary GF, and relapse) (7, 12). Poor graft function was defined as persistent cytopenia in at least two lineages (platelet < 20 × 10^9^/L, neutrophil count < 0.5 × 10^9^/L, hemoglobin level < 70 g/L) and/or requiring a transfusion beyond day +28, and full donor chimerism without relapse or severe GVHD (21). The Diagnosis and GVHD grade was based on the established criteria (22, 23). During the follow-up, the recipient’s BM was checked monthly for three months and every three to six months for the following one to two years.

### HRQoL Evaluation

Patients were excluded who had survived less than one year after transplantation, were less than 14 years old at the time of the questionnaire survey, had experienced relapse or GF, experienced any mental disorder after transplantation, or were unwilling to participate in the quality of life survey. A survey packet was mailed to every patient willing to complete a questionnaire that included a consent form, a set of questionnaires concerning their HRQoL, and a self-addressed stamped envelope. Each respondent was asked to sign the consent form and complete the HRQoL questionnaires (at 18 months after transplantation) before returning these materials to the investigators at their earliest convenience.

The 36-Item Short-Form Health Survey (SF-36) included eight subscales: physical functioning, role-physical functioning, bodily pain, general health, vitality, social functioning, role-emotional functioning, and mental health. Raw scores were transformed into standardized scores on a scale of 0–100. High scores represented high function levels. The subscales were aggregated into two summary measures, physical components and mental components.

### Statistical Analysis

Because patient allocation in this study was based on the HLA-identical or HLA-haploidentical group assignments rather than by random assignment, the baseline levels of some clinical characteristics were imbalanced between the two groups. To reduce the influence of potential confounders, propensity score matching (PSM) was performed in this study. The propensity score that indicated the HLA status for each patient was calculated based on a multivariate logistic regression model. In this model, patient and donor age, female donor into male recipient, year of transplant (from January, 2014), disease status, and graft source between the two groups were used as covariates. Patients in the HLA-identical group were matched to those in the HLA-haploidentical group using 1:1 nearest neighbor matching with a caliper width of 0.2.

After PSM, 88 pairs of patients were created, and outcomes were compared between the two matched-pair groups. For demography, disease and treatment-related factors, and the SF-36 scores, the Mann-Whitney U test and the Pearson chi-squared test were used to compare continuous variables and categorical variables, respectively. Survival analysis was conducted using the Kaplan-Meier method and the log-rank test to compare differences. Engraftment and GVHD were estimated as cumulative incidences, considering early death as a competing event. Multivariate logistic regression and Cox proportional hazard regression analyses were applied to evaluate the contribution of independent factors. *P* < 0.05 was considered statistically significant.

The final date for follow-up for all surviving patients was August 31, 2020. SPSS 22.0 statistical software (IBM, Armonk, NY, USA) was used for statistical analysis.

## Results

### Patient Characteristics

As shown in **Table 1**, the proportion of vSAA at diagnosis was higher in the Haplo-cord HSCT group than the MRD-HSCT group (*P* = 0.001). The median recipient age was significantly lower in the Haplo-cord HSCT group than the MRD-HSCT group (*P* < 0.001). Similarly, the proportion of patients younger than 40 years in the Haplo-cord HSCT group was higher than the MRD-HSCT group. With respect to donors, the median age was significantly higher in the Haplo-cord HSCT group than the MRD-HSCT group (*P* < 0.001). Also, more male donors were included in the Haplo-cord HSCT group than the MRD-HSCT group (*P* = 0.013), which contributed to the difference in the donor-recipient sex match between the Haplo-cord HSCT and MRD-HSCT groups. The median follow-up time was longer in the MRD-HSCT group than that in the haplo-cord HSCT group (*P* = 0.003). All these variables were balanced between the Haplo-cord HSCT and MRD-HSCT groups after PSM (all *P* > 0.05) (**Table 1**).

**Table 1:** Patient and donor (graft) characteristics between the two groups.

Variables	*Before matching*			*After matching*		
	MRD-HSCT (n = 128)	Haplo-cord HSCT (n = 180)	*P*	MRD-HSCT (n = 88)	Haplo-cord HSCT (n = 88)	*P*
Median age, years (range)	29 (4–56)	24 (3–55)	**< 0.001**	29 (14–52)	29 (14–55)	0.901
Age, no. (%)			**0.004**			0.345
< 40 years	97 (75.8)	159 (88.3)		73 ( 83.0)	68 (77.3)	
≥ 40 years	31 (24.2)	21 (11.7)		15 (17.0)	20 (22.7)	
Gender (male/female), no.	70/58	105/75	0.524	48/40	48/40	1.000
Disease status (SAA/vSAA), no.	93/35	98/82	**0.001**	28/60	28/60	1.000
With PNH clone, no. (%)	13 (10.2)	21 (11.7)	0.677	10 (11.4)	11 (12.5)	0.816
ECOG score, median (range)	1 (0–2)	1 (0–2)	0.589	1 (0–2)	1 (0–2)	0.701
Previous transfusion						
Median units of RBC (range)	23 (3–36)	22 (2–38)	0.306	24 (4–36)	22 (2–36)	0.267
Median units of PLT (range)	22 (0–120)	18 (2–120)	0.067	22 (2–118)	20 (2–120)	0.098
Median SF, ng/mL (range)	1300 ( 248–4250 )	1680 (180–4550 )	0.059	1350 ( 278–4050 )	1610 (180–4352 )	0.072
Median time from diagnosis to HSCT, months (range)	3 (0.5–360)	2 (0.5–240)	0.440	5 (1–200)	2 (0.7–240)	0.987
HCT-CI			0.132			0.502
≤ 1	107 (83.6)	161 (89.4)		75 ( 85.2)	78 (88.6)	
≥ 2	21 (16.4)	18 (10.6)		13 (14.8)	10 (11.4)	
Unfront treatment, no. (%)	116 (90.4)	137 (76.1)	**0.001**	78 (88.6)	69 (78.4)	0.067
Donor median age, years (range)	31 (5–56)	41.5 (8–63)	**< 0.001**	32 (8–56)	31 (11–57)	0.742
Donor-recipient sex match, no. (%)			**0.009**			0.076
Male-male	28 (21.9)	69 (38.3)		20 (22.7)	31 (35.2)	
Male-female	32 (25.0)	41 (22.8)		24 (27.3)	18 (20.5)	
Female-male	42 (32.8)	36 (20.0)		28 (31.8)	17 (19.3)	
Female-female	26 (20.3)	34 (18.9)		16 (18.2)	22 (25.0)	
Donor sex, no. (%)			**0.013**			0.450
Male	60 (46.9)	110 (61.1)		44 (50.0)	49 (55.7)	
Female	68 (53.1)	70 (38.9)		44 (50.0)	39 (44.3)	
Blood types of donor to recipient, no. (%)			0.120			0.639
Matched	79 (62.0)	96 (53.0)		52 (59.1)	45 (51.1)	
Major mismatched	15 (11.6)	38 (21.5)		12 (13.6)	17 (19.3)	
Minor mismatched	24 (18.6)	37 (20.5)		18 (20.5)	21 (23.9)	
Major and minor mismatched	10 (7.8)	9 (5.0)		6 (6.8)	5 (5.7)	
Source of graft, no. (%)			**< 0.001**			0.089
BM	17 (13.3)	17 (9.4)		11 (12.5)	13 (14.8)	
PB	30 (23.4)	9 (5.0)		17 (19.3)	7 (8.0)	
BM + PB	81 (63.3)	154 (85.6)		60 (68.2)	68 (77.3)	
Median BM/PB MNCs, × 10^8^/kg (range)	11.0 (2.3–22.4)	11.2 (3.6–33.4)	0.308	10.8 (3.6–31.2)	10.7 (2.6–22.1)	0.301
Median BM/PB CD34^+^ cells, × 10^6^/kg (range)	3.8 (1.0–16.9)	3.6 (0.7–8.9)	0.342	3.7 (0.8–8.6)	3.9 (1.2–16.4)	0.351
Median cord TNCs, × 10^7^/kg (range)	–	2.1 (1.1–7.3)	–		2.0 (1.1–3.9)	–
Median cord CD34^+^ cells, × 10^5^/kg (range)	–	0.6 (0.1–2.3)	–		0.5 (0.1–2.3)	–
Median follow-up time, months (range)	51.5 (12.0–220.0)	39.0 (10.0–108.0)	**0.003**	53.0 (12.0–121.0)	48.0 (10.0–103.0)	0.134

Haplo-cord HSCT, haploidentical hematopoietic stem cell transplantation with unrelated cord blood infusion; MRD-HSCT, matched related donor hematopoietic stem cell transplantation; SAA, severe aplastic anemia; vSAA, very SAA; PNH, paroxysmal nocturnal haemoglobinuria; ECOG,eastern cooperative oncology group scale; RBC, red blood cell; PLT, paltelet; SF, serum ferritin; HCT-CI, hematopoietic stem cell transplantation-comorbidity index; BM, bone marrow; PB, peripheral blood; MNCs, mononuclear cells; TNCs, total nucleated cells.

The bold values were statistically significant (P < 0.05).

The two groups were matched with respect to the ratio of males to females of recipient, the time from diagnosis to transplantation, and other characteristics whether before or after PSM.

### Engraftment

85 of 88 patients in the Haplo-cord HSCT group survived more than +28 days. Among the 85 patients, two experienced primary GF, and the remaining 83 patients achieved successful HID engraftment without mixed chimerism of unrelated CB. In the MRD-HSCT group, 83 of 88 patients survived more than +28 days, and the 83 patients achieved successful MRD engraftment. The cumulative incidences of neutrophil engraftment +28 days between the Haplo-cord HSCT and MRD-HSCT were not different (97.7 ± 1.6% *vs* 100.0 ± 0.0%, *P* = 0.497), and the cumulative incidence of platelet engraftment +60 days between them were also similar (96.5 ± 2.0% *vs* 96.2 ± 2.1%, *P* = 0.804) (**Table 2**).

**Table 2:** Transplantation-related events between the two groups.

Variables	MRD-HSCT (n = 88)	Haplo-cord HSCT (n = 88)	*P*
Cumulative incidence of neutrophil engraftment +28 days (%)	100 ± 0.0	97.7 ± 1.6	0.497
Cumulative incidence of platelet engraftment +60 days (%)	96.2 ± 2.1	96.5 ± 2.0	0.804
Median days to ANC > 0.5 × 10^9^/L (range)	11 (8–23)	12 (9–27)	0.001
Median days to PLT > 20.0 × 10^9^/L (range)	13 (8–80)	15 (9–210)	**0.003**
Primary GF, no. (%)	0 (0.0)	2 (2.3)	0.477
Secondary GF, no. (%)	1 (1.1)	2 (2.3)	1.000
Infection, no. (%)	47 (53.4)	48 (54.5)	0.880
Relapse, no. (%)	0 (0.0)	0 (0.0)	1.000
Early death, no. (%)	8 (11.0)	8 (11.0)	1.000
TRM, no. (%)	20 (22.7)	16 (18.2)	0.455
Primary GF, no. (% of TRM)	0 (0.0)	1 (6.3)	0.444
Secondary GF, no. (% of TRM)	0 (0.0)	1 (6.3)	0.444
aGVHD, no. (% of TRM)	1 (5.0)	5 (31.3)	0.069
cGVHD, no. (% of TRM)	1 (5.0)	1 (6.3)	1.000
Infection, no. (% of TRM)	10 (50.0)	4 (25.0)	0.176
TA-TMA, no. (% of TRM)	4 (20.0)	2 (12.5)	0.672
Intracranial hemorrhage, no. (% of TRM)	2 (10.0)	1 (6.3)	1.000
MDS, no. (% of TRM)	0 (0.0)	0 (0.0)	–
PTLD, no. (% of TRM)	1 (5.0)	0 (0.0)	1.000
Poor graft function, no. (% of TRM)	1 (5.0)	1 (6.3)	1.000

Haplo-cord HSCT, haploidentical hematopoietic stem cell transplantation with unrelated cord blood infusion; MRD-HSCT, matched related donor hematopoietic stem cell transplantation; ANC, absolute neutrophil count; PLT, platelet; TRM, transplantation related mortality; aGVHD, acute graft-versus-host-disease, cGVHD, chronic GVHD; GF, graft failure; MDS, myelodysplastic syndrome; TA-TMA, transplantation-associated thrombotic microangiopathies; PTLD, post-transplant lymphoproliferative disease.

The bold values were statistically significant (P < 0.05).

The median time to achieve neutrophil engraftment in the Haplo-cord HSCT and MRD-HSCT groups was 12 days and 11 days, respectively, which was significantly different (*P* = 0.001) (**Table 2**). The median time to achieve platelet engraftment in the Haplo-cord HSCT and MRD-HSCT groups was 15 days and 13 days, respectively, which was significantly different (*P* = 0.003) (**Table 2**). Based on multivariate analysis, Haplo-cord HSCT was the only unfavorable factor that affected the median time to achieve neutrophil and platelet engraftment (*P* = 0.004; *P* = 0.001, respectively) (**Table 4**).

As of the last follow-up, except for two patients with secondary GF, sustained full donor chimerism was observed in the surviving patients after the Haplo-cord HSCT. One patient experienced secondary GF in the MRD-HSCT group, and mixed chimerism was detected in another patient at 9 months after transplantation. However, this patient was restored to full donor chimerism after infusion of MSCs and donor lymphocytes.

### GVHD Incidence and Severity

**Figure 1:**
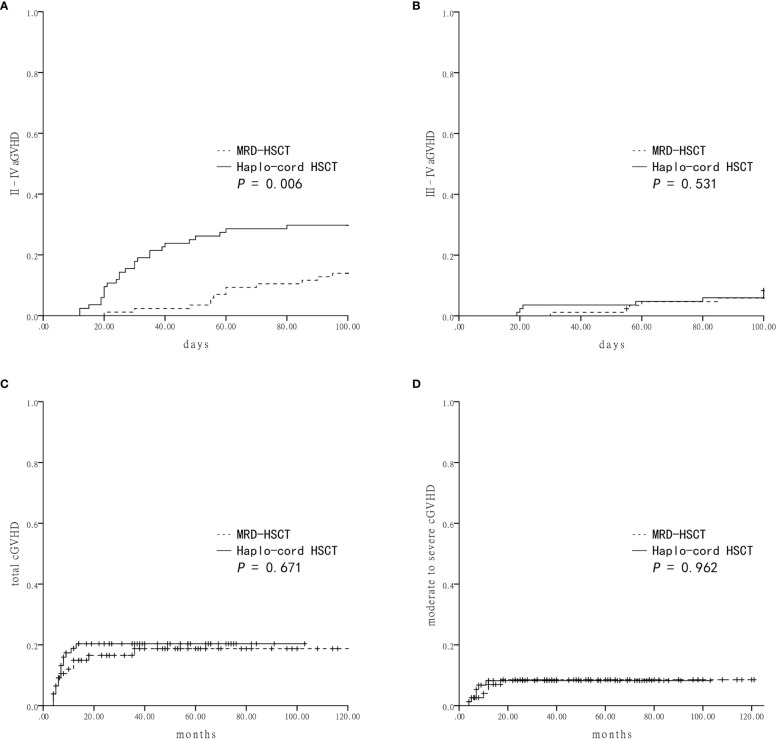
Graft-versus-host-disease (GVHD) after Haplo-cord HSCT or MRD-HSCT **(A)** The cumulative incidence of grade II–IV acute GVHD (aGVHD). **(B)** The cumulative incidence of grade III–IV aGVHD. **(C)** The cumulative incidence of total chronic GVHD (cGVHD). **(D)** The cumulative incidence of moderate to severe cGVHD.

The cumulative incidence of grade II–IV aGVHD within 100 days in the Haplo-cord HSCT group was significantly higher than the MRD-HSCT group (29.8 ± 5.0% *vs* 14.0 ± 3.7%, *P* = 0.006) (**Figure 1A**). However, the cumulative incidence of grade III–IV aGVHD was not different between the Haplo-cord HSCT and the MRD-HSCT groups (8.3 ± 3.0% *vs* 5.9 ± 2.5%, *P* = 0.531) (**Figure 1B**). Multivariate analysis identified Haplo-cord HSCT as the only unfavorable factor for II–IV aGVHD (*P* = 0.014) (**Table 4**).

Patients who lived longer than 100 days after transplantation were evaluated for the cumulative incidence of cGVHD. There were no differences in the total cGVHD between the Haplo-cord HSCT and the MRD-HSCT groups (20.4 ± 4.7% *vs* 18.7 ± 4.8%, *P* = 0.671, **Figure 1C**), and in the moderate to severe cGVHD between them (8.2 ± 3.2% *vs* 8.5 ± 3.3%, *P* = 0.962, **Figure 1D**). Multivariate analysis identified no significant factor in the total or moderate to severe cGVHD (**Table 4**).

### TRM

There was no patient with relapse in our study. The cumulative rate of transplant-related mortality between the Haplo-cord HSCT and the MRD-HSCT groups was not significantly different (18.2% *vs* 22.7%, *P* = 0.455). In the Haplo-cord HSCT group, four patients (25.0%) died from an infection, and six patients (37.6%) died from GVHD (five from aGVHD and one from cGVHD). In the MRD-HSCT group, ten patients (50.0%) died from infection, and two patients (10.0%) died from GVHD (one from aGVHD and one from cGVHD). Additional details of transplant-related events were shown in **Table 2**.

### Survival

**Figure 2:**
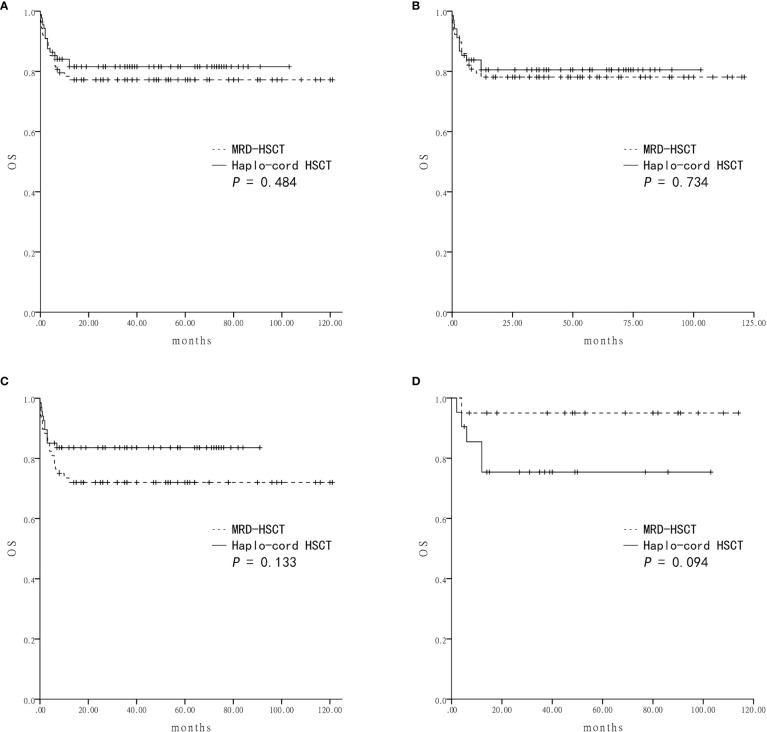
The estimated overall survival (OS) at four-year based on donor source **(A)** The OS was 81.5 ± 4.2% in Haplo-cord HSCT and 77.2 ± 4.5% in MRD-HSCT groups as a whole. **(B)** The OS was 80.5 ± 4.9% in Haplo-cord HSCT and 78.1 ± 4.7% in MRD-HSCT subgroups as the first-line treatment. **(C)** The OS was 83.6 ± 4.5% in Haplo-cord HSCT and 72.0 ± 5.5% in MRD-HSCT subgroups with patients aged < 40 years. **(D)** The OS was 75.4 ± 9.6% in Haplo-cord HSCT and 95.0 ± 4.9% in MRD-HSCT subgroups with patients aged ≥ 40 years.

**Figure 3:**
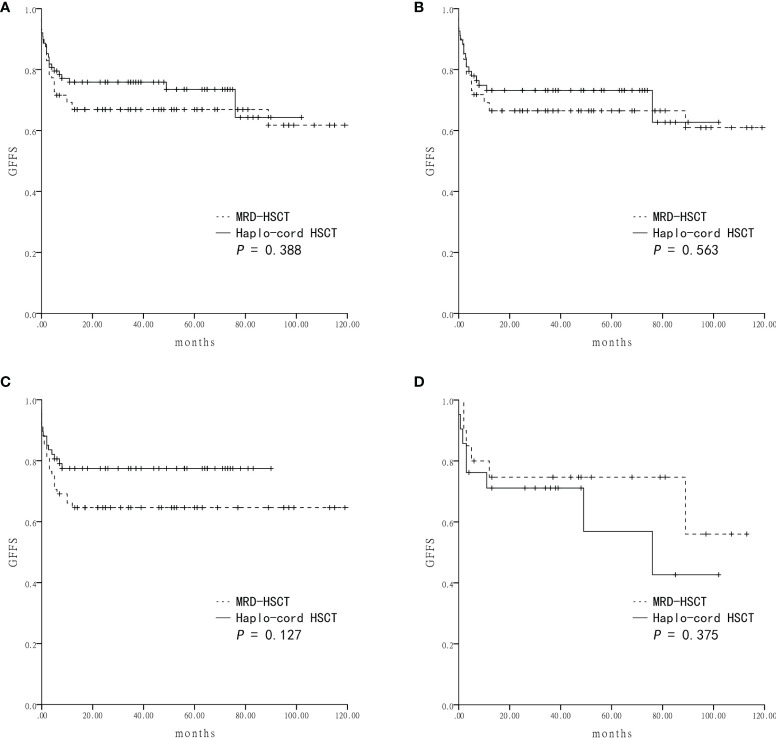
The estimated GVHD-free and failure-free survival (GFFS) at four-years based on the donor source **(A)** The GFFS was 73.5 ± 5.0% in Haplo-cord HSCT and 66.9 ± 5.0% in MRD-HSCT groups as a whole. **(B)** The GFFS was 62.7 ± 10.7% in Haplo-cord HSCT and 60.9 ± 7.2% in MRD-HSCT subgroups as the first-line treatment. **(C)** The GFFS was 77.5 ± 5.1% in Haplo-cord HSCT and 64.6 ± 5.8% in MRD-HSCT subgroups with patients aged < 40 years. **(D)** The GFFS was 42.7 ± 16.6% in Haplo-cord HSCT and 56.0 ± 17.8% in MRD-HSCT subgroups with patients aged ≥ 40 years.

Survival was assessed four years after transplantation. The estimated OS was similar between the Haplo-cord HSCT group and the MRD-HSCT group (81.5 ± 4.2% *vs* 77.2 ± 4.5%, *P* = 0.484) (**Figure 2A**). The estimated GFFS was also similar between the two group (73.5 ± 5.0% *vs* 66.9 ± 5.0%, *P* = 0.388) (**Figure 3A**).

Subsequent subgroup analysis showed that for patients receiving HSCT as first-line treatment, the estimated OS rates were similar between the Haplo-cord HSCT group (n = 68) and the MRD-HSCT group (n = 78) (80.5 ± 4.9% *vs* 78.1 ± 4.7%, *P* = 0.734) (**Figure 2B**). The estimated GFFS rates were also similar (62.7 ± 10.7% *vs* 60.9 ± 7.2%, *P* = 0.563) (**Figure 3B**).

Next subgroup comparisons between Haplo-cord HSCT and MRD-HSCT was performed according to the patient age. Among patient less than 40 years of age, OS and GFFS tended to be better in the Haplo-cord HSCT group (n = 67) compared with the MRD-HSCT group (n = 68), although it did not reach statistical significance (OS: 83.6 ± 4.5% *vs* 72.0 ± 5.5%%; *P* = 0.133; and GFFS: 77.5 ± 5.1% *vs* 64.6 ± 5.8%; *P* = 0.127) (**Figures 2C** and **3C**. Nevertheless among patient 40 years and older, OS and GFFS tended to be higher in the MRD-HSCT group (n = 20) compared with the Haplo-cord HSCT group (n = 21), although it did not reach statistical significance (OS: 95.0 ± 4.9% *vs* 75.4 ± 9.6%; *P* = 0.094; and GFFS: 56.0 ± 17.8% *vs* 42.7 ± 16.6. %; *P* = 0.375) (**Figures 2D** and **3D**), possibly due to the small sample size. Multivariate analysis identified no significant factors that were associated with OS and GFFS (**Table 4**).

### SF-36 Scores

The scores were higher for the physical component summary, physical functioning, general health, and social functioning in the Haplo-cord HSCT group than that in the MRD-HSCT group (all *P* < 0.05). No significant differences were observed for bodily pain, role-physical functioning, mental component summary, vitality, role-emotional functioning, and mental health between the two groups (all *P* > 0.05) (**Table 3**).

**Table 3:** HRQoL measures of the survivors between the two groups.

SF-36 scores (IQR)	MRD-HSCT(n = 45)	Haplo-cord HSCT(n = 49)	*P*
Physical			
Physical component summary	79.3 (69.3–83.8)	84.3 (76.1–91.7 )	**0.002**
Physical functioning	90.0 (80.0–95.0)	95.0 (90.0–95.0)	**0.001**
Role-physical functioning	75.0 (50.0–75.0)	75.0 (50.0–100.0)	0.096
Bodily pain	100.0 (100.0–100.0)	100.0 (95.5–100.0)	0.170
General health	57.0 (42.0–67.5)	67.0 (62.0–72.0)	**< 0.001**
Psychological			
Mental component summary	90.1 (84.4–92.4)	90.0 (84.9–96.4)	0.233
Vitality	85.0 (75.0–90.0)	85.0 (77.5–90.0)	0.651
Social functioning	87.5 (75.0–87.5)	100.0 (87.5–100.0)	**< 0.001**
Role-emotional functioning	100.0 (100.0–100.0)	100.0 (100.0–100.0)	0.950
Mental health	88.0 (84.0–92.0)	88.0 (82.0–88.0)	0.147

Haplo-cord HSCT, haploidentical hematopoietic stem cell transplantation with unrelated cord blood infusion; MRD-HSCT, matched related donor hematopoietic stem cell transplantation; HRQoL, health-related quality of life; IQR, interquartile range.

The bold values were statistically significant (P < 0.05).

In the multivariate analysis, moderate to severe cGVHD was one adverse risk factor associated with general health, vitality, and social functioning (all *P* < 0.05) (**Table 4**). Younger patient at transplantation was a favorable factor for role-physical functioning, bodily pain, vitality, social functioning, role-emotional functioning, mental health, physical component summary, and mental component summary (all *P* < 0.05), and the choice of Haplo-cord HSCT was another favorable factor for physical functioning, general health, social functioning, and mental health (all *P* < 0.05) (**Table 4**).

**Table 4:** Multivariate analysis of favorable factors associated with outcomes.

Outcomes	OR/HR (95% CI)	*P*
Median days to ANC > 0.5 × 10^9^/L		
Group (**Haplo-cord HSCT** *vs* MRD-HSCT)	0.758 (0.607–0.892)	**0.004**
Median days to PLT > 20.0 × 10^9^/L		
Group (**Haplo-cord HSCT** *vs* MRD-HSCT)	0.524 (0.324–0.958)	**0.001**
Grade II–IV aGVHD		
Group ( **Haplo-cord HSCT** *vs* MRD-HSCT)	2.613 (1.212–5.636)	**0.014**
Grade III–IV aGVHD		
Group (**Haplo-cord HSCT** *vs* MRD-HSCT)	0.919 (0.378–2.234)	0.853
Total cGVHD		
Group (**Haplo-cord HSCT** *vs* MRD-HSCT)	1.210 (0.533–2.747)	0.649
Moderate-severe cGVHD		
Group (**Haplo-cord HSCT** *vs* MRD-HSCT)	0.974 (0.519–1.829)	0.936
Overall survival		
Group (**Haplo-cord HSCT** *vs* MRD-HSCT)	0.792 (0.410–1.528)	0.487
GVHD-free/failure-free survival		
Group (**Haplo-cord HSCT** *vs* MRD-HSCT)	0.790 (0.457–1.363)	0.397
Physical functioning		
Group (**Haplo-cord HSCT** *vs* MRD-HSCT)	3.436 (1.402–8.422)	**0.007**
Patient age	0.977 (0.934–1.023)	0.329
Moderate to severe cGVHD	0.963 (0.390–2.378)	0.936
Role-physical functioning		
Group (**Haplo-cord HSCT** *vs* MRD-HSCT )	1.373 (0.560–3.370)	0.489
Patient age	0.944 (0.900–0.990)	**0.018**
Moderate to severe cGVHD	1.615 (0.655–3.981)	0.298
Bodily pain		
Group (**Haplo-cord HSCT** *vs* MRD-HSCT )	0.447 (0.128–1.562)	0.207
Patient age	0.896 (0.835–0.962)	**0.002**
Moderate to severe cGVHD	0.579 (0.164–2.050)	0.397
General health		
Group (**Haplo-cord HSCT** *vs* MRD-HSCT)	5.760 (2.223–14.921)	**0.001**
Patient age	0.974 (0.928–1.021)	0.275
Moderate to severe cGVHD	2.593 (1.005–6.688)	**0.047**
Vitality		
Group (**Haplo-cord HSCT** *vs* MRD-HSCT)	1.789 (0.703–4.555)	0.222
Patient age	0.945 (0.899–0.992)	**0.023**
Moderate to severe cGVHD	2.516 (0.987–6.412)	**0.043**
Social functioning		
Group (**Haplo-cord HSCT** *vs* MRD-HSCT)	7.250 (2.544–20.666)	**0.001**
Patient age	0.923 (0.875–0.974)	**0.003**
Moderate to severe cGVHD	3.697 (1.316–10.385)	**0.013**
Role-emotional functioning		
Group (**Haplo-cord HSCT** *vs* MRD-HSCT)	0.823 (0.251–2.701)	0.748
Patient age	0.925 (0.867–0.988)	**0.019**
Moderate to severe cGVHD	1.065 (0.323–3.511)	0.918
Mental health		
Group (**Haplo-cord HSCT** *vs* MRD-HSCT)	0.353 (0.144–0.869)	**0.023**
Patient age	0.949 (0.905–0.995)	**0.029**
Moderate to severe cGVHD	1.960 (0.804–4.779)	0.139
Physical component summary		
Group (**Haplo-cord HSCT** *vs* MRD-HSCT)	1.950 (0.783–4.857)	0.152
Patient age	0.929 (0.885–0.976)	**0.003**
Moderate to severe cGVHD	1.959 (0.784–4.896)	0.150
Mental component summary		
Group (**Haplo-cord HSCT** *vs* MRD-HSCT)	1.430 (0.548–3.730)	0.465
Patient age	0.925 (0.878–0.975)	**0.003**
Moderate to severe cGVHD	2.001 (0.764–5.242)	0.158

OR, odds ratio; HR, hazard risk; CI, confidence interva; vs, versus; Haplo-cord HSCT, haploidentical hematopoietic stem cell transplantation with unrelated cord blood infusion; ANC, absolute neutrophil count; PLT, platelet; aGVHD, acute graft-versus-host-disease; cGVHD, chronic graft-versus-host-disease.

The bold values were statistically significant (P < 0.05).

## Discussion

This multicenter study was conducted to comprehensively compare the outcomes of large SAA patients cohort underwent Haplo-cord HSCT or MRD-HSCT. First of all, similar rate of hematopoietic engraftment was observed in the Haplo-cord HSCT and MRD-HSCT groups. Nevertheless, engraftment speed of neutrophil and platelet favored the MRD-HSCT group, which meant that the Haplo-cord HSCT might need more supportive care. These outcomes were similar to another study (24). GVHD was a common complication after engraftment, and it might be directly related to survival and quality of life of the survivors, especially in severe cases. In this study, although higher proportions of grade II–IV aGVHD was observed in the Haplo-cord HSCT group than the MRD-HSCT group, similar cumulative incidences were observed in grade III–IV aGVHD, total cGVHD, and moderate to severe cGVHD. Meanwhile, no differences in the aGVHD and cGVHD-related TRM were found between the two groups. The multivariate analysis determined that grade II–IV aGVHD was related to Haplo-cord HSCT. The following factors likely contributed to explain the accepted GF and GVHD in the Haplo-cord recipients. First, adequate CD34+ cells were present, including mobilized BM and PB from the HIDs. Second, additional immunosuppressant was administered due to higher incidences of aGVHD in this group.

Next, we compared the survival of SAA patients after Haplo-cord HSCT or MRD-HSCT. In general, the OS and GFFS rates were comparable between the two groups, including between the corresponding Haplo-cord HSCT and MRD-HSCT as a first-line treatment for SAA patients, which was consistent with another study (7). Considering that old age was determined to be a strong negative predictor in SAA patients receiving allo-HSCT (25), we performed subgroup analyses with the age of 40 as the cut-off. Among patients younger than 40 year, at least comparable OS and GFFS rates were observed between Haplo-cord HSCT and MRD-HSCT groups. Therefore, it was feasible to recommend the Haplo-cord HSCT for SAA patients without MRDs, which was consistent with another report (26). Patients older than 40 years had a significantly poorer prognosis in the Haplo-cord HSCT than that in the MRD-HSCT group, nevertheless, the differences were not statistically significant, probably because the number of patients in the over 40 years old subgroup were too small to draw a persuasive conclusion. Despite these limitations, our data supported 2015 edition of the British Guide for SAA, the age limit for HLA-identical HSCT in SAA patients was expanded to 50 years (3). Until recently, Yang et al. reported that the combination of MRD-HSCT with an unrelated CB unit could achieve favorable outcomes in SAA patients aged 35 to 50 years (27). Another study reported comparable survival outcomes of transplantation from HIDs and matched donors for SAA patients aged 40 years and older (28). Of course, it is necessary to perform further studies with a large sample size to confirm these outcomes of Haplo-cord HSCT for SAA patients aged 40 years and older and explore some risk factors.

With high survival rates in SAA patients with transplantation, quality of life concerns are considered equally important by physicians and patients (29). Our results suggested that scores from most subscales for physical health and social functioning subscale for psychological health were higher in SAA patients undergoing the Haplo-cord HSCT than the MRD-HSCT, while the other subscales’ scores were similar between the two groups. Although similar comparative results also were reported by Mo et al. (30), our study was the first to make the comparison specifically for SAA and not multiple blood diseases. Recovery of HRQoL after allo-HSCT in most survivors is a complicated process requiring three to five years and is influenced by many factors, including age, sex, transplant type, later complications, time after transplantation, and many others (31, 32). Another study about HRQoL reported that mild and moderate cGVHD was significantly better than severe cGVHD, and patients with moderate cGVHD without multiple organ involvement and more severe organ impairment were better off than patients who experienced these conditions (33). In our multivariate analysis, moderate to severe cGVHD was a negative factor that affected most physical and psychological HRQoL of the survivors. Fortunately, the incidence of moderate to severe cGVHD was similar between the Haplo-cord HSCT and the MRD-HSCT groups. In accordance with other studies (31, 34), we also observed that a younger age was associated with a higher score for physical and psychological HRQoL. Because the median recipients’ age after PSM at the time of transplantation was not different between the Haplo-cord HSCT and the MRD-HSCT groups, illustrating the effect of age on the difference in HRQoL between the two groups was limited in my study. In this case, Haplo-cord HSCT, a favorable factor of part subscales, can make a great contribution to a comparable to better physiological quality of life in the Haplo-cord HSCT group than the MRD-HSCT group. Therefore, long-term SAA survivors receiving Haplo-cord HSCT can attain desirable HRQOL comparable to better than that of patients receiving MRD-HSCT.

One limitation in this study is that some survivors did not reply to our invitation. A response rate greater than 70% is low but is not unreasonable for these cross-sectional studies. This low response rate may be related to our failure to offer a reward and to design a face-to-face questionnaire. Another disadvantage of this study is that retrospective HRQoL scores suffered from recall bias, and this phenomenon may be overcome by performing further perspective studies.

In summary, Haplo-cord HSCT for SAA patients exhibited several interesting results compared to the MRD-HSCT, (1), relatively slower engraftments of the neutrophil and platelet, (2), higher incidences of aGVHD, while similar moderate to severe cGVHD, (3), similar OS and GFFS between the whole group and the corresponding subgroups, and (4), comparable to better HRQoL. These outcomes supported the recommendation that Haplo-cord HSCT should be considered an effective alternative option for SAA patients who lack a MRD. However, this result should be supported further by a well-designed, prospective study.

## Data Availability Statement

The original contributions presented in the study are included in the article/supplementary material. Further inquiries can be directed to the corresponding authors.

## Ethics Statement

Written informed consent was obtained from the individual(s), and minor(s)’ legal guardian/next of kin, for the publication of any potentially identifiable images or data included in this article.

## Author Contributions

M-QL, X-LL, and Y-MZ wrote the manuscript and performed the analysis. D-PW, MM, and L-ML designed the protocol. All authors contributed patients, provided clinical and laboratory data, and revised and corrected the manuscript.

## Funding

This work was partially supported by grants from the National Natural Science Foundation of China (81730003), National Science and Technology Major Project (2017ZX09304021), National Key R&D Program of China (2019YFC0840604, 2017YFA0104502), Priority Academic Program Development (PAPD) of Jiangsu Higher Education Institutions, Jiangsu Medical Outstanding Talents Project (JCRCA2016002). Jiangsu Provincial Key Medical Center (YXZXA2016002). The Natural Science Foundation of Jiangsu Province (Grants No.BK20180202).

## Conflict of Interest

The authors declare that the research was conducted in the absence of any commercial or financial relationships that could be construed as a potential conflict of interest.

## Publisher’s Note

All claims expressed in this article are solely those of the authors and do not necessarily represent those of their affiliated organizations, or those of the publisher, the editors and the reviewers. Any product that may be evaluated in this article, or claim that may be made by its manufacturer, is not guaranteed or endorsed by the publisher.
